# Propylthiouracil Attenuates Experimental Pulmonary Hypertension via Suppression of Pen-2, a Key Component of Gamma-Secretase

**DOI:** 10.1371/journal.pone.0137426

**Published:** 2015-09-14

**Authors:** Ying-Ju Lai, Gwo-Jyh Chang, Yung-Hsin Yeh, Jong-Hwei S. Pang, Chung-Chi Huang, Wei-Jan Chen

**Affiliations:** 1 Department of Respiratory Therapy, Chang Gung University College of Medicine, Chang-Gung University, Tao-Yuan, Taiwan; 2 Graduate Institute of Clinical Medical Sciences, Chang Gung University College of Medicine, Chang-Gung University, Tao-Yuan, Taiwan; 3 Cardiovascular Division, Chang Gung Memorial Hospital, Tao-Yuan, Taiwan; 4 Division of Thoracic Medicine, Chang Gung Memorial Hospital, Tao-Yuan, Taiwan; Georgia Regents University, UNITED STATES

## Abstract

Gamma-secretase-mediated Notch3 signaling is involved in smooth muscle cell (SMC) hyper-activity and proliferation leading to pulmonary arterial hypertension (PAH). In addition, Propylthiouracil (PTU), beyond its anti-thyroid action, has suppressive effects on atherosclerosis and PAH. Here, we investigated the possible involvement of gamma-secretase-mediated Notch3 signaling in PTU-inhibited PAH. In rats with monocrotaline-induced PAH, PTU therapy improved pulmonary arterial hypertrophy and hemodynamics. *In vitro*, treatment of PASMCs from monocrotaline-treated rats with PTU inhibited their proliferation and migration. Immunocyto, histochemistry, and western blot showed that PTU treatment attenuated the activation of Notch3 signaling in PASMCs from monocrotaline-treated rats, which was mediated via inhibition of gamma-secretase expression especially its presenilin enhancer 2 (Pen-2) subunit. Furthermore, over-expression of Pen-2 in PASMCs from control rats increased the capacity of migration, whereas knockdown of Pen-2 with its respective siRNA in PASMCs from monocrotaline-treated rats had an opposite effect. Transfection of PASMCs from monocrotaline-treated rats with Pen-2 siRNA blocked the inhibitory effect of PTU on PASMC proliferation and migration, reflecting the crucial role of Pen-2 in PTU effect. We present a novel cell-signaling paradigm in which overexpression of Pen-2 is essential for experimental pulmonary arterial hypertension to promote motility and growth of smooth muscle cells. Propylthiouracil attenuates experimental PAH via suppression of the gamma-secretase-mediated Notch3 signaling especially its presenilin enhancer 2 (Pen-2) subunit. These findings provide a deep insight into the pathogenesis of PAH and a novel therapeutic strategy.

## Introduction

Pulmonary arterial hypertension (PAH) is characterized by narrowing and obstruction of small pulmonary arteries with resulting in increased pulmonary vascular resistance and right ventricular hypertrophy, which may originate from dysfunction of pulmonary arterial smooth muscle cells (PASMCs) [1, 2]. The contribution of signaling pathways, such as prostacyclin [3], endothelin [4], serotonin [5], platelet-derived growth factor [6, 7], and Notch3 [8], to these pathological processes has been the subject of intensive investigation.

A prior study has demonstrated Notch3 to be a crucial pathway for PASMC dysfunction and PAH development [8]. The Notch receptor (Notch1-4) is an integral-membrane protein and can be activated by gamma-secretase cleavage to an intracellular domain (ICD) and the succeeding translocation into the nucleus [9]. Within the nucleus, Notch ICD acts as a transcript factor and regulates its downstream targets, such as hairy and enhancer-of-split (Hes), to affect SMC function [10,11]. Gamma-secretase is a protease complex in the cellular membrane consisting of presenilin 1 and 2 (PSEN1 and 2), nicastrin, anterior pharynx-defective 1 (Aph-1), and presenilin enhancer 2 (Pen-2) subunits [12,13]. Among these, nicastrin and Aph-1 stabilize Pen-2 and induce endoproteolysis of presenillin [13–15]. Conceivably, inhibition of gamma-secretase function may block the activation of Notch3 signaling and have a potential effect on PASMC dysfunction and PAH formation.

Propylthiouracil (PTU), a widely used drug for hyperthyroidism, possesses an anti-thyroid effect by inhibiting iodide oxidation, monoiodotyrosine iodination, and coupling steps in thyroxine (T4) production, as well as the peripheral conversion of T4 to triiodothyronine (T3) [16]. Beyond its anti-thyroid effect, our previous studies show that PTU also inhibits atherosclerosis/neointimal formation in aortas of rabbits fed a high cholesterol diet and balloon-injured rat carotid arteries through a thyroid-independent action [17,18]. These effects are attributed to its effects on either inhibiting vascular smooth muscle cell proliferation and migration or promoting differentiation and endothelium-dependent vasodilatation [17–20]. Because there are similar patho-mechanisms between atherosclerosis and PAH, another group also demonstrates PTU to have an inhibitory effect on PAH development [21,22]. However, detailed mechanisms underlying PTU-inhibited PAH formation remained unresolved. The aim of this study is, therefore, to evaluate mechanisms behind the suppressive effect of PTU on PAH, especially focusing on the underlying signaling pathway responsible for PAH.

## Materials and Methods

### Ethics statement

This study was carried out in strict accordance with the recommendations in the Guide for the Care and Use of Laboratory Animals of the National Institutes of Health. All animal experiments were reviewed and approved the study protocols by the Institutional Animal Care and Use Committee of Chang Gung University (Permit Number: CGU11-067). All the surgery was performed under ketamine and xylazine i.p. anesthesia, and all efforts were made to minimize suffering. Housing and maintenance was provided by Chang Gung University, All animals were fed a standard chow diet with free access to water.

### Monocrotaline-treated rats

Adult male Sprague-Dawley rats (200–250g body weight) underwent either single subcutaneous injection of 60 mg/kg monocrotaline (MCT) (Sigma-Aldrich) or control saline alone. Fourteen days later, MCT-treated rats were divided into 3 treatment groups: 1) PTU group receiving PTU (5 mg/100g/day) (Sigma-Aldrich) daily by gavage for 14 days; 2) PTU/T3 group receiving PTU plus 10 μg/100g/day T3 (Sigma-Aldrich; dissolved in 2.5 mmol/L NaOH) intramuscularly; 3) control group receiving water only. The T3 dose was designed to normalize thyroid hormone levels [17].

### Hemodynamic measurements and cardiovascular evaluation

For monitoring hemodynamics, rats were anesthetized with ketamine and xylazine i.p. Hemodynamic data were obtained at the 28th day after MCT injection. The right carotid artery was cannulated, and a 1.6F catheter tipped pressure transducer (Scisense, Canada) was inserted through the right jugular vein for measuring right ventricular systolic pressure (RVSP). After sacrifice, left lung was fixed for histology in 10% neutral buffered formalin, and right lung was snap-frozen in liquid nitrogen. For the assessment of RV hypertrophy, the RV was separated from the left ventricular (LV) wall and ventricular septum. Wet weight of the RV and free LV wall with ventricular septum was determined. RV hypertrophy was expressed as the ratio of RV wall and LV free wall plus ventricular septum (LV+S).

### Immunohistochemical analysis

Immunohistochemical analysis of lung tissues was performed with primary antibodies against α-smooth muscle (SM)-actin (Sigma-Aldrich) and Notch3 ICD (Abcam) using the Dako LSAB peroxidase kit (Dako). Staining of α-SM-actin was used to indicate the medial layer of small pulmonary arteries (PAs) for the assessment of medical wall thickness (MWT). For the Notch3 signal, lung tissue sections were incubated with rabbit anti-Notch3 and mouse anti-α-SM-actin antibodies for 1 hour, followed by incubation with Alexa-488-conjugated secondary antibody (green, Invitrogen) for Notch3 or Cy3-conjugated secondary antibody (red, Chemicon) for α-SM-actin at room temperature for 30 minutes, and observed with a Leica TCS SP spectral confocal microscope at Microscope Core Laboratory of Chang Gung Memorial Hospital.

### Assessment of MWT

The percentage of MWT was used to represent medial hypertrophy by α-SM-actin staining. Under 400X microscopic examination, MWT was defined as the distance between the internal and external elastic laminae using “image J” software from http://rsb.info.nih.gov/ij/download.html. For vascular sections, the diameter was defined as (longest diameter + shortest diameter)/2. Each group included 20–25 slides under 400X microscopic examination [23].

### Cell culture

Rat PASMCs were isolated from lungs of MCT-treated or control Sprague-Dawley rats using explant method as described previously [7,24,25]. Rats were initially anesthetized with ketamine and xylazine i.p. To obtain distal PASMCs (smaller than 100 μm), the main PA was dissected free from lung and cardiac tissues using a single full-length incision and flashed with Hank’s balanced salt solution (Gibco, Invitrogen). The distal arterial tissue was cut into small pieces and resuspended in Dulbecco’s modified Eagle’s medium (DMEM) (Gibco, Invitrogen) containing 100 U/mL penicillin, 100 g/mL streptomycin (PAN Biotech, Aidenbach, Germany), and 20% fetal bovine serum (FBS) (Gibco, Invitrogen) and subsequently cultured in 10 cm plate and incubated at 37°C in 5%CO_2_/95%air. After 24 hours, the medium was changed and thereafter every 2–3 days. Characterization of PASMCs was determined by immunocytochemical staining with anti-α-SM-actin (Sigma-Aldrich) and anti-desmin (NeoMakers) antibodies. PASMCs with passages before 3 were used in all *in vitro* experiments [24]. PTU was dissolved in 100% dimethysulphoxide (DMSO) at final concentrations of 1∼10 mmol/L. Final concentration of DMSO in the culture medium was less than 0.1%. Cells were treated with 0.1%DMSO as a vehicle.

### Hypoxia treatment

PASMCs from control rats were cultured in the hypoxic condition for 24 hours to determine the effect of PTU or DAPT (Calbiochem) on the Notch3 and gamma-secretase subunit expressions. Hypoxia was created in an incubator: 5% CO2+94% N2+10% O2, 37°C. DAPT were dissolved in 100% DMSO at a final concentration of 10 mmol/L.

### Notch-specific ligand treatment

PASMCs from control rats were serum-starved for 48 hours, then cultured in Jagged-1 peptide (a selective Notch-specific ligand) in combination with PTU or DAPT for 24 hours to determine the effect of PTU or DAPT on the Notch3 and gamma-secretase subunit expressions. The active fragment of Jag-1 protein (aa 188–204; AnaSpec, Fremont, CA) and the scrambled peptide with a random sequence of the amino acids were added at the same concentration (1 micromol/L).

### Western blot analysis

For western blotting, immunoblotting was performed using anti-Notch3 ICD (Abcam), anti-Hes5 (Santa Cruz Biotech), and anti-gamma-secretase subunits (PSEN1, 2, nicastrin, Aph-1, and Pen-2) (Cell signaling) as primary antibodies. Secondary antibodies were specific for peroxidase conjugated anti-mouse IgG or anti-rabbit IgG (Sigma-Aldrich) as needed. Blots were visualized using the enhanced chemiluminescence detection system (Amersham). Samples were normalized to GAPDH (Cell signaling) or lamin B (Abcam) and quantified by densitometry.

### Immunocytochemical analysis

Immunocytochemical analysis in PASMCs was performed with primary antibodies against α-SM-actin (Sigma-Aldrich), Notch3 ICD (Abcam), proliferating cell nuclear antigen (PCNA) (Sigma-Aldrich), and gamma-secretase subunits (Cell signaling). At the end of experiments, cells were rinsed with PBS, fixed with cold methanol for 5 minutes at room temperature; after removal of methanol; washed twice with 1xPBS, blocked with 1%goat serum/1%BSA in PBS for 30 minutes, and incubated with primary antibodies for 1 hour. Following that, cells were incubated with Alexa-488-conjugated (green) and Cy3-conjugated (red) secondary antibodies for gamma-secretase subunit and α-SM-actin signaling at room temperature for 30 minutes. Nuclei were visualized by DAPI-staining (Gibco, Invitrogen). Fluorescence was observed with a confocal microscope at Microscope Core Laboratory of Chang Gung Memorial Hospital.

### Cell proliferation assay

The proliferative activity of PASMC was determined by 5-bromo-2-deoxyuridine (BrdU) incorporation assay using an ELISA detecting kit (Roche Diagnostics Co.,) following the manufacturer’s instructions.

### Cell migration assay

Transwell filter chamber (Corning Costar) with 8.0 μm pore size was used for migration assay. PASMCs were seeded at a density of 5×10^5^ cells per filter. To initiate the chemotaxis assay, cells in 200 μL DMEM without FBS were added to the upper chamber and the bottom chamber was filled with 600 μL DMEM plus 10% FBS as chemotaxis factor for cell moving. PASMCs were allowed to migrate at 37°C for 6 hours. Cells on the lower aspect of filter membrane were stained with Liu's stain. Total filter membrane was divided to 6 fields. Each field was randomly photographed and counted [17].

### Small interfering (si) RNA

Chemically synthetic siRNA for Pen-2 and its control siRNA were purchased from Dharmacon (Dharmacon/Thermo Fisher Scientific) and transferred into PASMCs using RNAiMax (Invitrogen) according to the manufacturer’s instructions.

### Plasmid construction and transfection

A Pen-2 expression vector was generated by PCR using primers: forward (5'-CCGAAGCTTATGAACTTAGAGCG-3') and reverse (5'-CTTTCTAGATTGGGAGTGCCC-3') (GenBank accession no. NM_001008764.2) and subcloned into the pcDNA3.1 vector at the HindIII/XbaI restriction sites. PASMCs grown to 70–80% confluence were transfected with indicated plasmids using Lipofectamine 2000 (Invitrogen) according to the manufacturer’s instructions. The transfection efficiency by this method was approximately 60%.

### Statistical analysis

Mean and standard error (SE) were used to describe the data. Differences between two groups were determined by unpaired *t*-test. For multiple groups, one-way ANOVA with post hoc bonferroni’s test was used to compare data among groups. A value of P≤0.05 was considered to be statistically significant.

## Results

### Effect of PTU on hemodynamic and structural changes in PAs

**Fig 1:**
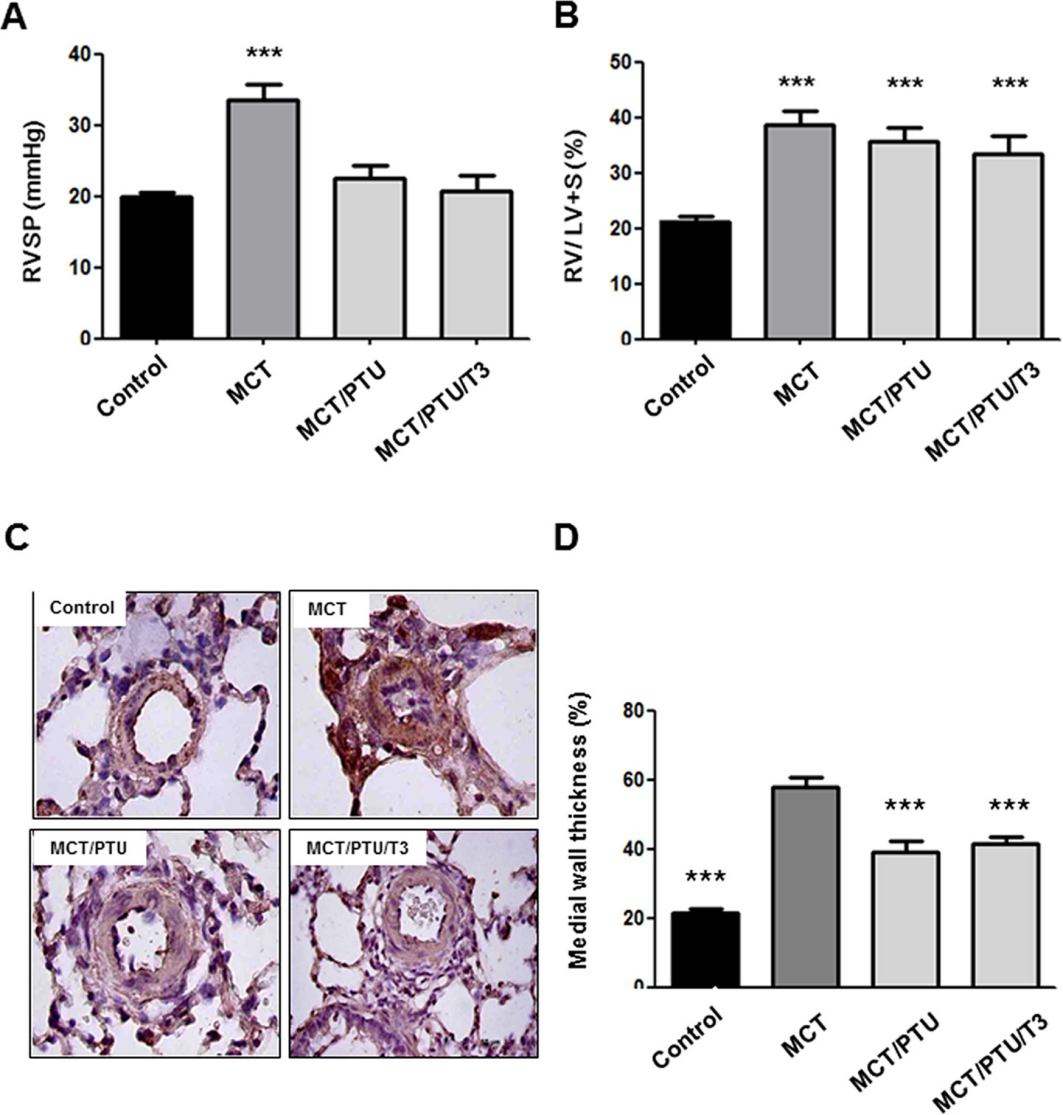
Effect of PTU on pulmonary hemodynamics and pulmonary arterial hypertrophy. (A) Right ventricle systolic pressure (RVSP) in 4 different groups is shown. Pulmonary hypertension (indicated by elevated RVSP) was established 28 days after MCT injection and PTU treatment (5 mg/100g/day by gavage from day 14 to 28) reduced RVSP in MCT-treated rats. (B) Ratio of RV to LV plus septum weight (RV/LV+S) is shown. (C) Medial wall thickness of small pulmonary arteries (25–50μm) identified by α-SM-actin staining (brown staining) is shown. (D) The degree of medial wall thickness was compared among 4 groups. Each value (mean±SE [n = 6–7]) is expressed. ***p<0.001 versus control or MCT group, one-way ANOVA, bonferroni's post-test.

Table 1 displays the values of 26 rats in 4 groups. There was a trend toward decrease in heart rate at the end of experiment in MCT/PTU group, possibly due to the anti-thyroid effect of PTU. As expected, rats challenged with MCT indeed developed PAH and RV hypertrophy, as indicated by increased RVSP and RV/LV+S weight ratio, respectively, at the 28th day after MCT injection compared with controls (Fig 1A). Administration of PTU from the 14th to 28th day after MCT injection reduced RVSP in MCT-treated rats. Nevertheless, PTU did not reverse RV/LV+S weight ratio, possibly due to the irreversible effect of MCT-induced RV hypertrophy (Fig 1B). Concomitant supplementation of PTU with T3 did not alter its suppressive effect on PAH development in MCT-treated rats, which precluded the involvement of its anti-thyroid effect in this process (Fig 1A). The MCT/PTU group exhibited a significant reduction of serum T3 level compared with control, MCT, and MCT/PTU/T3 groups during sacrifice (Table 1). In small PA wall (25–50μm), the MWT increased in MCT group and, mutually, the luminal diameter of small PA decreased compared with controls (Fig 1C and 1D). In MCT/PTU and MCT/PTU/T3 groups, PTU treatment resulted in a significant reduction in the MWT of small PA compared with MCT group (Fig 1D), which was compatible with hemodynamic findings.

**Table 1:** Characteristics of the experimental study groups.

	Control	MCT	MCT/PTU	MCT/PTU/T3
**Animal (n)**	7	7	6	6
**Weight (g) at sacrifice**	377.3±30.0	306.7±43.2	282.3±9.3	344.3±12.5
**Heart rate (bpm) at sacrifice**	371.3±6.4	336.7±11.0	271.0±9.2✝	398.7±11.1*
**T3 level (ng/dL) at sacrifice**	46.9±13.94	42.5±9.55	28.6±6.18ǂ	44.9±8.3

MCT = monocrotaline; PTU = propylthiouracil; PA = pulmonary artery; T3 = triiodothyronine

All data are presented as mean ± SE; *p-value<0.05, ^✝^p-value<0.01, ǂp-value<0.001versus MCT

### Effect of PTU on PASMC proliferation and migration

**Fig 2:**
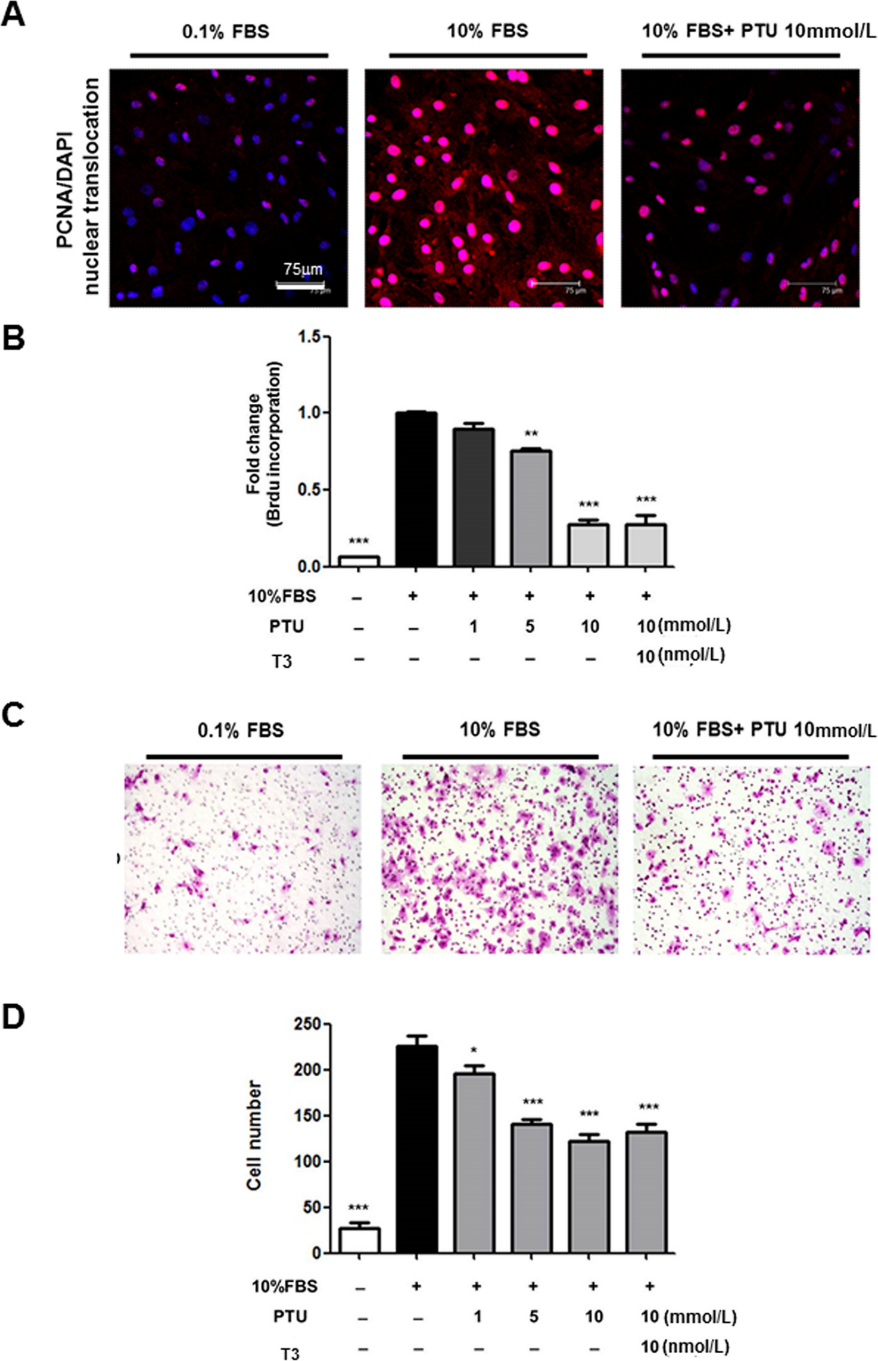
Effect of PTU on serum-induced PASMC proliferation and migration. After 24 hours of serum deprivation, PASMCs from MCT rats were treated with indicated conditions for 24 hours. Translocation of proliferating cell nuclear antigen (PCNA, red staining; scale bar: 75μm) into the nucleus **(A)** and BrdU incorporation **(B)** were used to represent proliferative activities of PASMCs. Each value (mean±SE [n = 6]) is expressed as fold change of BrdU incorporation in control cells with 0.1% FBS. (**C)** Migratory activity of PASMC was assessed by migration assay chamber. After treatment with indicated conditions for 6 hours, PASMCs from MCT rats at the lower aspect of filter membrane was fixed and stained with Liu’s stain (pink staining; magnification 100X). (**D)** Each value (mean±SE [n = 6]) is determined by cell number at the lower aspect of filter membrane, and expressed as a percentage of control. *p<0.05, **p<0.01, ***p<0.001 versus 10% FBS without PTU, one-way ANOVA, bonferroni’s post test.

Proliferation and migration of PASMCs contribute greatly to PAH development [1,2]. The next experiment was designed to evaluate whether the inhibitory effect of PTU on PAH formation *in vivo* could be applied *in vitro*. Treatment of cultured PASMCs with PTU led to a concentration-dependent decrease in serum-induced PASMC proliferation (Fig 2A and 2B) and migration. Furthermore, exogenous supplementation of T3 in PTU-treated cells did not abrogate PTU effects (Fig 2C and 2D), giving further evidence that these effects are independent of its anti-thyroid and extra-thyroidal effects.

### Effect of PTU on Notch3 expression in proximal and distal PAs

**Fig 3:**
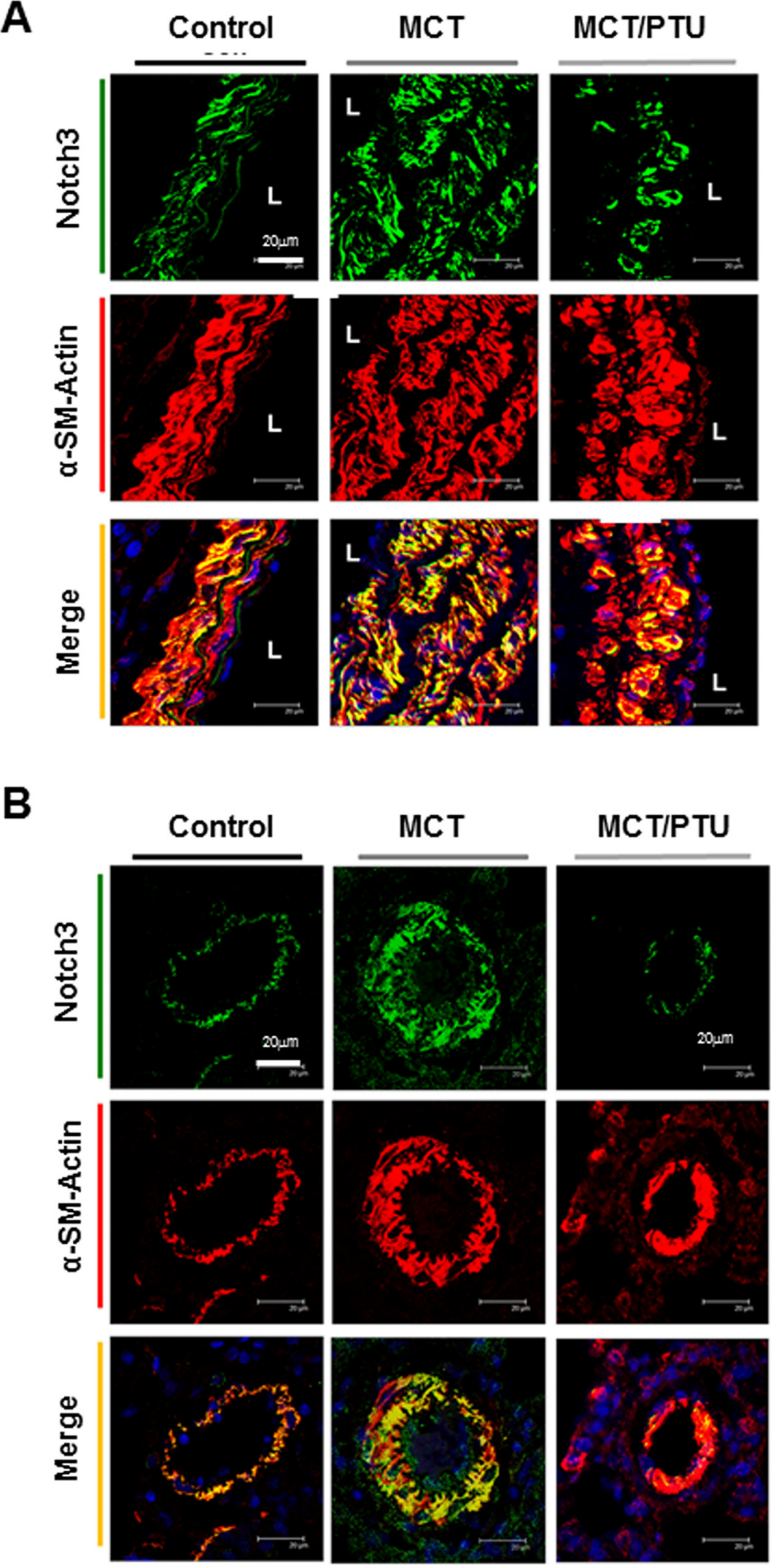
Effect of PTU on Notch3 and gamma-secretase subunit expression in proximal and distal pulmonary arteries. Immuohistochemistry shows Notch3 expression in the proximal part (A) (scale bar: 20μm; L: vascular lumen) and the distal part of pulmonary arteries (B) (scale bar: 20μm). Notch3 in medial layer of pulmonary arteries was identified by α-SM-actin double staining for vascular SMC. The picture is a representative of 4 independent experiments.

Notch3, a member of Notch family, is associated with SMC proliferation and differentiation [10,11]. Li and colleagues demonstrate that Notch3 also plays a crucial role in PAH pathogenesis [8]. In consistent with their findings, immunohistochemistry showed that MCT rats displayed a higher expression of Notch3 (identified by co-localization with α-SM-actin) in the medial layer of proximal and distal PAs than control rats (Fig 3). The expression of Notch3 was lower in the PAs of PTU-treated MCT rats than in those of MCT rats (Fig 3A and 3B)**.** Notably, there was a gradual decrease in Notch3 expression from lumen to media/adventitia in the proximal PAs of PTU-treated rats (Fig 3A), possibly reflecting that PTU effect may originate for lumen.

### Effect of PTU on Notch3 activation in PASMCs

**Fig 4:**
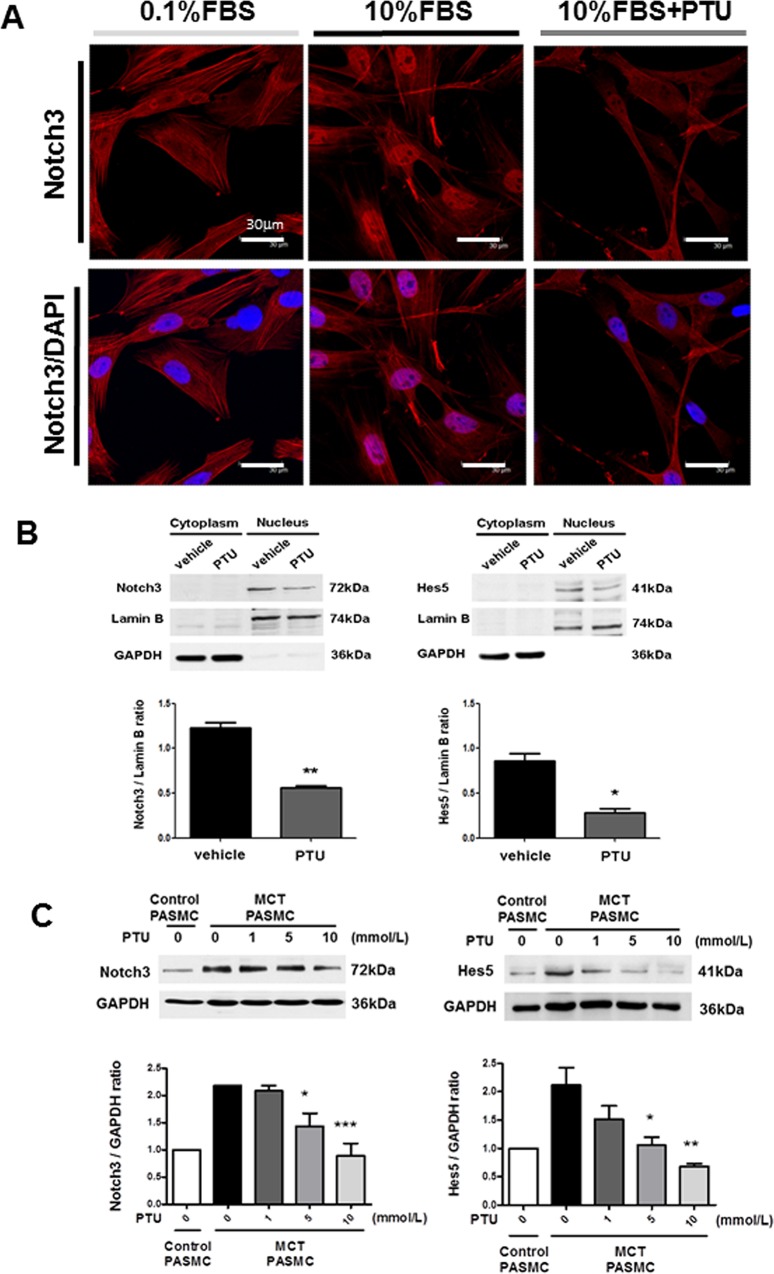
Effect of PTU on Notch3 activation in cultured PASMCs. **(A)** After 24 hours of serum deprivation, PASMCs from MCT rats were treated with indicated conditions for 6 hours. Immunocytochemistry shows Notch3 ICD (red staining) localization (nuclear staining with DAPI in blue and Notch/DAPI merging in pink; scale bar: 30μm). The picture is a representative of 4 independent experiments. **(B)** After 24 hours of serum deprivation, PASMCs from MCT rats were treated with or without 10mmol/L PTU for 6 hours. Western blot shows Notch3 ICD and Hes5 expression in nuclear extraction. The relative expression level of each protein is quantified by densitometry and normalized to lamin B. Each value represents the mean±SE of 4 independent experiments. *P<0.05, **p<0.01 versus control PASMC. (**C)** After 24 hours of serum deprivation, PASMCs from control and MCT rats were treated with indicated conditions for 24 hours. Western blot shows Notch3 ICD and Hes5 expression in whole cell extraction. The relative expression level of each protein is quantified by densitometry and normalized to GAPDH. Each value represents the mean±SE of 4 independent experiments. *P<0.05, **p<0.01, ***p<0.001 versus control PASMCs without PTU, one-way ANOVA, bonferroni’s post test

Notch3 signaling pathway is activated by gamma-secretase cleavage and the ensuing release of its ICD fragments into the nucleus [9]. To further confirm the critical role of Notch3 in PTU effects, we evaluated the effect of PTU on Notch3 activation. First of all, immunocytochemistry showed that PTU inhibited serum-induced nuclear translocation of Notch3 ICD in cultured PASMCs from MCT rats (Fig 4A). Furthermore, western blot verified the decreased Notch3 ICD and its key downstream target (Hes5) expression in the nuclear extract of PTU-treated PASMCs (Fig 4B). Finally, western blot analysis using whole cell lysates also showed that PTU treatment concentration-dependently down-regulated Notch3 ICD and Hes5 in PASMCs from MCT rats (Fig 4C). In agreement with *in vivo* findings, PASMCs from MCT rats still expressed a higher level of Notch3 ICD and Hes5 than those from control rats (Fig 4C). Taken together, these findings demonstrate that PTU may inhibit Notch3 activation in PASMCs from PAH rats.

### Effect of PTU on gamma-secretase expression

**Fig 5:**
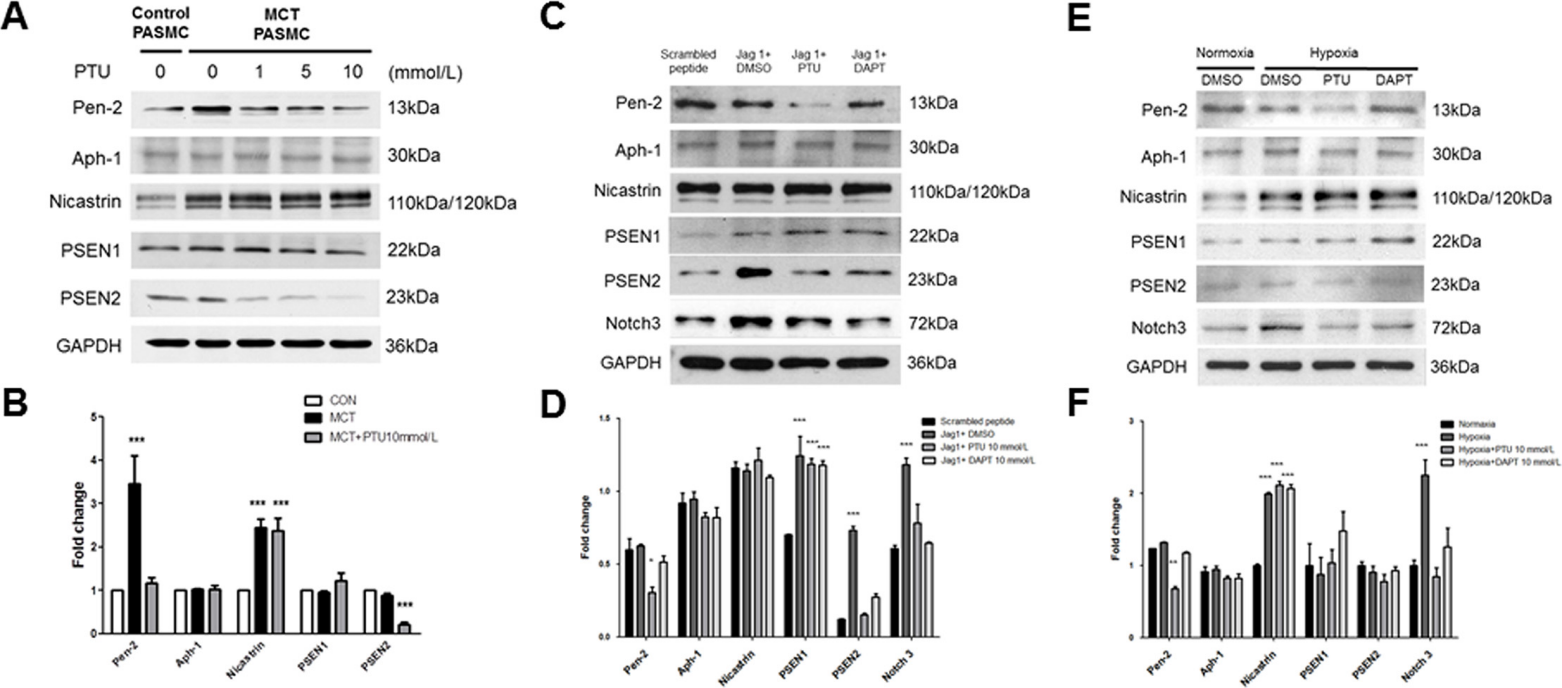
Effect of PTU on gamma-secretase subunit expression in cultured PASMCs. (A, C) After 24 hours of serum deprivation, PASMCs from control or MCT rats were treated with indicated conditions for 24 hours. Western blot shows gamma-secretase subunit expression in whole cell extraction. (B, D, F) The relative expression level of each protein is quantified by densitometry and normalized to GAPDH. (E) PASMCs from control rats were treated with indicated conditions for 24 hours. Western blot shows gamma-secretase subunit expression in whole cell extraction. Each value represents the mean±SE of 4 independent experiments. *P<0.05, **p<0.01, ***p<0.001 versus control PASMCs without PTU or scrambled peptide or Normaxia, one-way ANOVA, bonferroni’s post test.

Because PTU may be involved in Notch3 activation, the next experiments were designed to investigate whether gamma-secretase, an enzyme essential for Notch3 activation [9], also contributes to PTU effect. Western blot revealed that PASMCs from MCT rats exhibited a higher expression of Pen-2, a key gamma-secretase subunit [12–15], than those from control rats (Fig 5A). Treatment of PASMCs from MCT rats with PTU reduced Pen-2 and PSEN2, 2 main gamma-secretase subunits, expression in a concentration-dependent manner (Fig 5B). Furthermore, we utilized a Notch-specific ligand (the active fragment of Jagged-1 protein) to test the effect of PTU on PASMCs. Western blot showed that Jagged-1 peptide stimulation up-regulated Notch3 and gamma-secretase subunits (PSEN 1 and 2) in PASMCs from control rats (Fig 5C and 5D). Concurrent administration of PTU in Jagged-1-treated control PASMCs reduced Notch3, Pen-2 and PSEN2 expressions (Fig 5C and 5D). Treatment of PASMCs from control rats with DAPT, a well-established Notch inhibitor, down-regulated Notch3 and PSEN2, but not Pen-2, suggesting the specific effect of PTU on inhibiting Pen-2. In addition, we applied an *in vitro* hypoxic (1% oxygen) model to further evaluate the effect of PTU on PASMCs. We found that PTU, but not DAPT, treatment in control PASMCs could decrease Pen-2 expression under an *in vitro* hypoxic condition (Fig 5E and 5F). Other subunits remained unaffected by PTU treatment (Fig 5), implicating that PTU could only affect the functional components of gamma-secretase. Together, these data suggest that the inhibitory effect of PTU on Notch3 activation from MCT PASMCs and from Jag-1 pathway as well as from hypoxic condition in control PASMCs might be mediated via Pen-2, a crucial gamma-secretase subunit.

### Role of Pen-2 in PASMC functions

**Fig 6:**
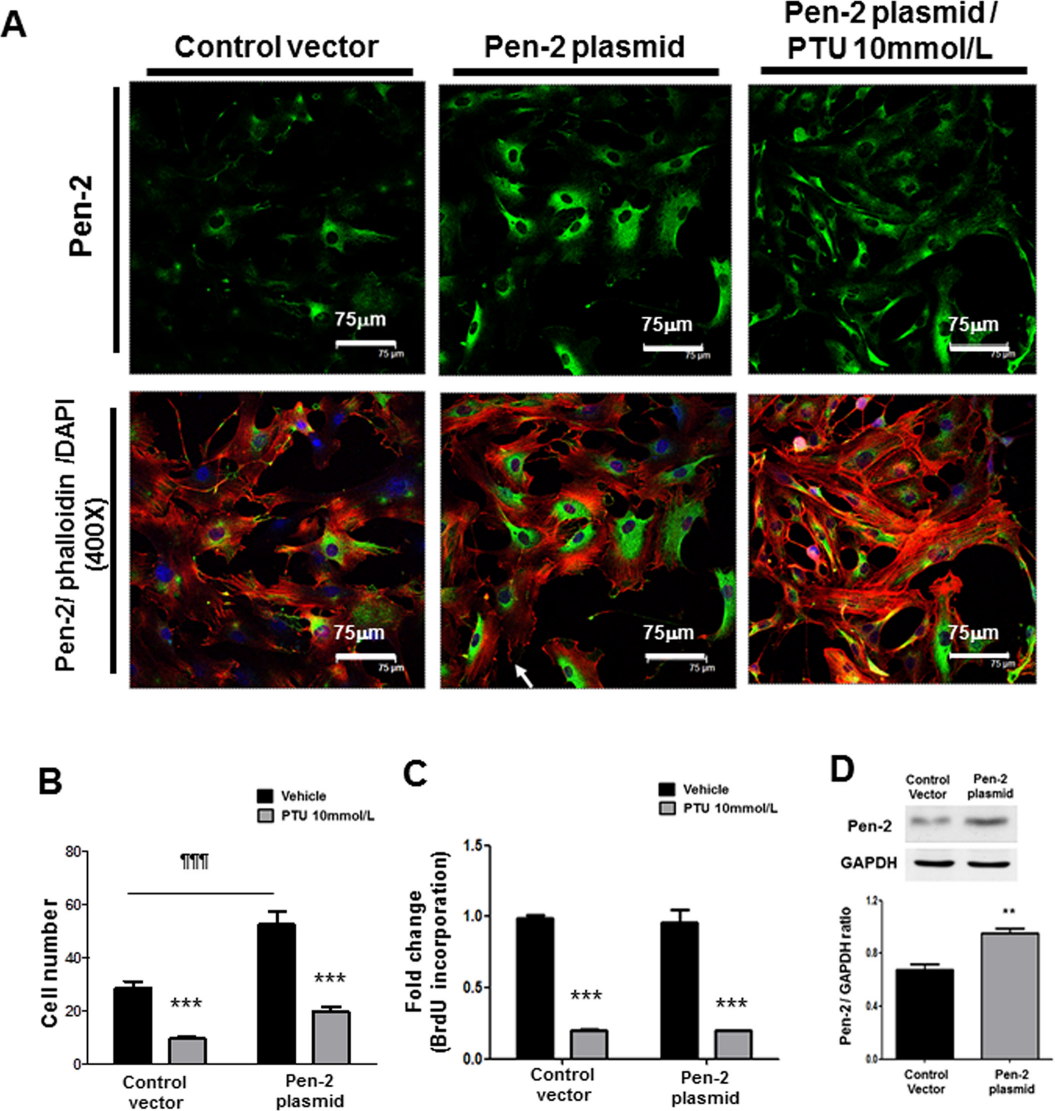
Effect of Pen-2 over-expression on PASMC proliferation and migration. **(A)** After transfection with indicated plasmids for 24 hours, PASMCs from control rats were treated with or without 10mmol/L PTU for 24 hours. Immunocytochemistry shows Pen-2 localization (green staining; nuclear staining with DAPI in blue, rhodamine-phalloidin staining in red, and Pen-2/phalloidin merging in yellow; scale bar: 75 and 20μm). The picture is a representative of 4 independent experiments. After transfection with indicated plasmids for 24 hours, PASMCs from control rats were treated with or without 10mmol/L PTU for 24 hours. Migration chamber assay **(B)** and BrdU incorporation **(C)** were used to assess migratory and proliferative activities of PASMCs, respectively. Each value represents the mean±SE of 4 independent experiments. ***p<0.001 PTU versus vehicle; ¶¶¶ p<0.001 Pen-2/vehicle versus control vector/vehicle. (**D)** After transfection with indicated plasmids for 24 hours, western blot shows over-expression of Pen-2 in PASMCs from control rats. Lower panel: The relative expression level of each protein is quantified by densitometry and normalized to GAPDH. Each value represents the mean ± SE of 4 independent experiments. **p<0.01 versus control vector

**Fig 7:**
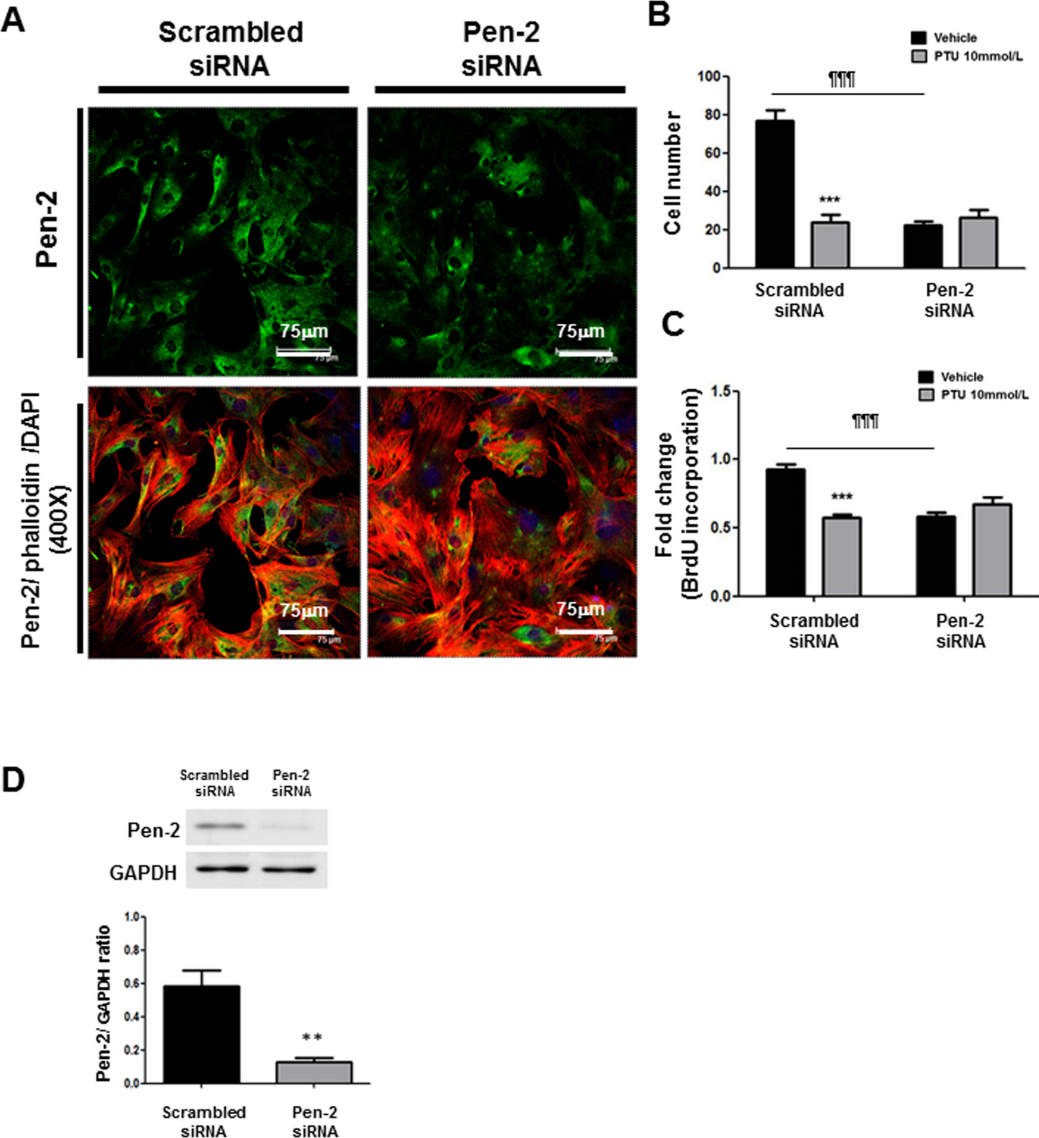
Effect of Pen-2 knock-down on PASMC proliferation and migration. **(A)** After transfection with indicated siRNAs in PASMCs from MCT rats for 24 hours, immunocytochemistry shown Pen-2 localization (green staining; nuclear staining with DAPI in blue, rhodamine-phalloidin staining in red, and Pen-2/phalloidin merging in yellow; scale bar: 75μm). The picture is a representative of 4 independent experiments. After transfection with indicated siRNAs for 24 hours, PASMCs from MCT rats were treated with or without 10mmol/L PTU for 24 hours. Migration chamber assay **(B)** and BrdU incorporation **(C)** were used to assess migratory and proliferative activities of PASMCs, respectively. Each value represents the mean±SE of 4 independent experiments. ***p<0.001 PTU versus vehicle; ¶¶¶ p<0.001 Pen-2 siRNA versus scrambled siRNA. (**D)** After transfection with indicated siRNAs for 24 hours, western blot shows knock-down of Pen-2 in PASMCs from MCT rats. Lower panel: The relative expression level of each protein is quantified by densitometry and normalized to GAPDH. Each value represents the mean±SE of 4 independent experiments. **p<0.01 versus scrambled siRNA.

The association of Notch3 with SMC migration has been intensely investigated [10,11]. However, little is known about the contributing role of Pen2, a key gamma-secretase subunit, in SMC migration and proliferation. To examine the potential role of gamma-secretase, especially Pen-2, in PASMC functions, we utilized gain-of-function and loss-of-function studies with Pen-2 expression vector and its respective siRNA, respectively. Immunocytochemistry disclosed that PTU treatment inhibited the Pen-2, gamma-secretase subunits in PASMCs (Fig 6A). Over-expression of Pen-2 in PASMCs from control rats induced PASMC migration (Fig 6B). Nevertheless, Pen-2 over-expression did not further enhanced PASMC proliferation (Fig 6C), possibly reflecting the minor role of Pen-2 in PASMC proliferation. In contrast, knock-down of Pen-2 in PASMCs from MCT rats with its siRNA reduced PASMC proliferation, migration (Fig 7). The efficiency of transfection was verified by western blot (Figs 6D and 7D). These data support the crucial role of Pen-2 a key gamma-secretase subunit in PASMC functions, especially migration.

### Role of Pen-2 in PTU effects

We next further utilized gain-of-function and loss-of-function studies to elucidate the essential role of Pen-2 in PTU effects. PTU still had an inhibitory effect on PASMC proliferation, migration in PEN2 over-expressed cells (Figs 6 and 7). Contrarily, Pen-2 knock-down in PASMCs from MCT rats reversed PTU-inhibited PASMC proliferation and migration (Fig 7B and 7C). These findings provide further evidence that Pen-2 is essential for PTU function.

## Discussion

The main finding presented here is that PTU, besides its anti-thyroid and anti-atherosclerotic effects, also serves as a gamma-secretase inhibitor to impede Notch3 signaling and experimental PAH in rats. The suppressive effect of PTU on PAH is evidenced by the improvement of pulmonary hemodynamics and regression of PA hypertrophy and PASMC dysfunction in MCT-treated rats. This study follows our previous studies regarding the beneficial effect of PTU on vascular SMCs and further extends this knowledge to PASMCs. We also confirm that Notch3 signaling plays a crucial role in the pathogenesis of PAH and may be a key therapeutic target. Notably, we demonstrate that Pen-2, the key gamma-secretase subunit, is essential for proliferation and migration in PASMCs and requisite for PTU-inhibited PASMC proliferation and migration. The gamma-secretase-dependent effect of PTU on inhibiting PASMC proliferation and migration may contribute to its action on suppressing PAH. Our findings provide valuable information on PAH treatment.

Pulmonary arterial vasoconstriction and remodeling (mainly due to PASMC proliferation and migration) constitute the main feature of early PAH [1,2]. As the disease progresses, the pulmonary vascular bed may lose an ability to dilate and make PA remodeling crucial in the late stages of PAH [1, 2, 26]. To date, many therapeutic targets have been selected for PAH treatment, including calcium channel [27, 28], endothelin receptor [29], platelet-derived growth factor [7], phosphodiesterase 5 [30,31], prostacyclin, and prostanoids [32,33]. Nevertheless, most of them address pulmonary vasodilation as the main therapeutic function and little is proven (either experimental or clinical) that any of these drugs exert an anti-proliferative effect or even an action to reverse proliferative changes in Pas [34]. In the present and our previous studies, we demonstrate that PTU possesses the inhibitory effect on proliferation/migration in either vascular SMCs or PASMCs [17], which may have beneficial effects in suppressing atherosclerosis/neointimal formation and the current PAH development. In comparison with conventional drugs, PTU provides an additional action on inhibiting PASMC proliferation and migration to suppress PAH development.

Prior study has demonstrated that Notch3 is associated with the development of PAH [8]. Beyond the previously characterized Notch3 pathway, our study further demonstrates the importance of its regulatory enzyme (gamma-secretase) in the course of PAH. This conclusion is based on the fact that gamma-secretase, especially its functional subunit (Pen-2), up-regulated in PASMCs from MCT-treated PAH rats either *in vivo* or *in vitro*. Furthermore, we found that there was a high correlation between gamma-secretase Pen-2 subunit expression and PASMC proliferative and/or migratory activities in our loss-of-function and gain-of-function studies. It is conceivable that Pen-2 play an important role in gamma-secretase-regulated Notch3 signaling and may induce PASMC dysfunction and thus promote PAH.

In this study, we have obviously demonstrated that PTU is a gamma-secretase inhibitor by reducing the expression of its main Pen-2 subunit. Gamma-secretase is comprised of 5 functional subunits: PSEN1, 2, Pen-2, nicastrin, and Aph-1[12,13]. Among these, the association of Pen-2 with nicastrin and Aph-1 is required for PSEN1-induced endoproteolysis and gamma-secretase activation [12–15]. Although the exact function of Pen-2 still remained unresolved, some gain-of function and loss-of function studies either *in vivo* or *in vitro* have documented the critical role of Pen-2 in the activity of gamma-secretase [14,15, 35–38]. In addition to its action on Notch receptor cleavage, gamma-secretase is also responsible for cutting the transmembrane domain of amyloid β-protein precursor to form amyloid β-protein [39]. Several evidences suggest that amyloid β-protein may accumulate in the brain and contribute to the pathogenesis of Alzheimer’s disease [39]. Therefore, gamma-secretase is considered to be the principal therapeutic target of Alzheimer’s disease and indeed many gamma-secretase inhibitors have been under clinical trial to treat Alzheimer’s disease patients [40]. Conceivably, PTU may have a potential application for the treatment of Alzheimer’s disease by its inhibitory action on gamma-secretase.

PTU also possesses other actions that potentially contribute to its beneficial effect on PAH. First, we and others have demonstrated that PTU is capable of inducing nitric oxide production in rat aortas and Pas [19,20]. Because impairment of nitric oxide synthesis is involved in the pathogenesis of PAH [1,2], the favorable effect of PTU on PAH may, at least in part, be attributed to its nitric oxide inducing action. Second, PTU is used as an anti-inflammatory agent to treat Graves’ disease in clinical practice [41]. Because inflammation may participate in PAH pathogenesis [1,2], the contribution of this anti-inflammatory effect should also be considered. Third, PTU has been recognized as a potent antioxidant [42,43]. Whether PTU is associated with the oxidative process in PAH development merits further investigation. Finally, we have previously reported that PTU has a promoting effect on vascular SMC differentiation; a phenotypic transit exhibiting the quiescent behavior [18]. Nevertheless, our unpublished observation finds that PTU seems unable to bring about a similar change in PASMC differentiation, suggesting that PTU might operate different functions between the pulmonary and systemic vessels.

In this study, we have demonstrated a good *in vivo* relevance in rat PAH mode with *in vitro* conditions. The dose of PTU used in these rats exceeds the standard dose in humans with 300 to 600 mg daily [41]. Nevertheless, our previous studies have documented that the blood concentration of PTU in PTU-treated rats lies within the therapeutic range in humans (1 to 10 μg/mL; ≈0.006∼0.06 mmol/L) [41]. Conceivably, PTU may suppress PAH development during clinical application. The concentration of PTU (1–10 mmol/L) presented here to affect cultured PASMC functions is much higher than the blood concentration. Our explanation for this discrepancy is that it may just reflect the acute effect of PTU on cultured PASMCs.

Prior studies have indicated the linkage between thyroid dysfunction (generally accepted as hyperthyroidism) and PAH [44]. As an anti-thyroid drug, the contributing role of thyroid hormone in PTU effect should be taken into account. In this study, we have virtually excluded this possibility due to the fact that supplementation of thyroid hormone does not alter the effect of PTU either *in vivo* or *in vitro*. Nevertheless, as we described previously [17], whether maintaining the circulating or extracellular T3 levels to a normal range may convert intracellular hypothyroid status to the euthyroid status needs to be further addressed. To our knowledge, there still has no reliable method for the measurement of intracellular T3 level up to now [45].
